# Translational Research in Colorectal Cancer: Current Status and Future Perspectives of Multimodal Treatment Approach

**DOI:** 10.1155/2019/2010259

**Published:** 2019-03-24

**Authors:** Luigi Marano, Gianluca Pellino, Christos Kontovounisios, Valerio Celentano, Matteo Frasson

**Affiliations:** ^1^Department of Medicine, Surgery and Neurosciences, Unit of General Surgery and Surgical Oncology, University of Siena, Italy; ^2^Department of Advanced Medical and Surgical Sciences, Università degli Studi della Campania “Luigi Vanvitelli”, Naples, Italy; ^3^Department of Colorectal Surgery, Chelsea and Westminster Hospital, London, UK; ^4^Department of Surgery and Cancer, Imperial College, London, UK; ^5^University of Portsmouth, Portsmouth, UK; ^6^Portsmouth Hospitals NHS Trust, Portsmouth, UK; ^7^Colorectal Unit, Hospital Universitario y Politécnico “La Fe”, Valencia, Spain

Despite significant improvements in medical as well surgical technologies over the last two decades, colorectal malignancies remain a serious challenge to human health globally, representing one of the leading causes of cancer-related deaths worldwide.

High-quality research and advances in technology have contributed to the elucidation of molecular pathways underlying disease progression and have stimulated many clinical studies testing tailored managements. Similar to many other malignancies, colorectal cancer is a heterogeneous disease, making it a clinical challenge for optimization of treatment modalities in reducing the morbidity and mortality associated with this disease. Actually, it appears essential to classify tumors based on the underlying oncogenic pathways and to develop biological as well as genotype-based molecular therapies acting on individual tumors, redefining deeply the natural history of the colorectal cancer and establishing a new standard for diagnostic, stadiative, and therapeutic tools.

This special issue is devoted to current and emerging multimodal treatment approaches in colorectal cancer on the basis of bench to bedside philosophy, focusing on some selected topics that are both interesting and challenging: molecular biology, genetic and epigenetic factors, noninvasive molecular biomarkers, and metagenomic study on colorectal microbiota.

X. He and colleagues performed a retrospective analysis to investigate the impact of tumor location on survival outcomes in a total of 377,849 colon cancer patients. Furthermore, they also examined whether the prognostic role of cancer location was influenced by different groups of age, stage, year of diagnosis, and therapies. This clinical-based study demonstrated that left colon cancer was associated with better prognosis in terms of overall as well as cancer-specific survival. The data reported in this study provide us with new insights into the relationship between tumor location and survival outcomes, stimulating future studies to explore underlying mechanisms.

S. H. Kim et al. focused on the role of cyclooxygenase-2 (COX-2) expression on stage I to III primary colorectal cancer tissues and the impact on the biologic behavior of recurrent disease after curative resection. Interestingly, they found that the mean time to recurrence was significantly longer in the elevated expression group, concluding that COX-2 expression was an independent factor associated with late recurrence (>3 years after surgery) during the follow-up period after surgery. Therefore, the positive COX-2 patients should be considered candidates for more frequent testing after 3 years of follow-up and extend follow-up period longer than 5 years after surgery. This study will help us to identify optimal surveillance methods and follow-up intervals.

The ideal colorectal cancer biomarker should be easily and quantitatively measured, highly specific, and sensitive, as well as reliable and reproducible. It should be able to stratify between different risk-based populations, selecting patients who really need a second-line test (endoscopic and radiologic investigations). Ideally, this aim can be achieved with a noninvasive and inexpensive method, using easily available biological samples such as urine, breath, serum, and feces. G. Pellino and colleagues reviewed the role of the newer noninvasive or minimally invasive biomarkers of colorectal cancer with evidence from currently available literature. They discussed imaging and biomolecular diagnostics ranging from their potential usefulness to obtain early and less-invasive diagnosis to their potential implementation in the development of a bespoke treatment of colorectal cancer. This paper will provide us with a comprehensive knowledge on noninvasive biomarkers and their roles in colorectal cancer management.

Another paper from A. O. Alomair et al. is aimed at investigating microbiota in colorectal cancer patients by means of metagenomic studies. Their results indicated that the colorectal cancer cases had significant enrichment of eleven genera compared to those in the control group. The metagenomic sequencing showed that specific species, such as Fusobacterium nucleatum, Peptostreptococcus stomatis, and Parvimonas micra, were present in significantly greater quantities in the CRC patients than those in the controls. The data reported in this study provide us with new insights into the relationship between microbiome alterations and susceptibility of colorectal cancer, suggesting strategies for early diagnosis, preventive measures, and curative therapies.

C. Bucci et al. provided a systematic review and a meta-analysis on current knowledge on the same-day bowel preparation prior colonoscopy. They proved that the same-day regimen is equivalent to the split preparation in terms of colon cleaning ability, on equal compliance and less sleep disturbance, giving to the clinicians the evidence to recommend this when the split preparation is unfeasible or does not fit the patients' needs.

In summary, the contributions of this special issue could stimulate the spread of novel molecular targets in colorectal malignancies and share some strategies to optimize diagnostic and curative approaches.

